# The Efficacy of Parent-Child Interaction Therapy (PCIT) in Children with Attention Problems, Hyperactivity, and Impulsivity in Dubai

**DOI:** 10.1155/2021/5588612

**Published:** 2021-03-04

**Authors:** Saad A. Al Sehli, Maya Helou, Meshal A. Sultan

**Affiliations:** ^1^Department of Psychiatry, Taibah University, Madinah, Saudi Arabia; ^2^Mental Health Centre of Excellence, Al Jalila Children's Specialty Hospital, Dubai, UAE; ^3^College of Medicine, Mohammed Bin Rashid University of Medicine and Health Sciences, Dubai, UAE

## Abstract

Disruptive behaviors can be associated with significant functional impairment. Early intervention for young children is essential to prevent long-term consequences. Parent-Child Interaction Therapy (PCIT) is a psychotherapeutic intervention, which has shown to be effective for children with externalizing symptoms. We present the treatment course of PCIT for two kindergarten children. The first has Attention-Deficit/Hyperactivity Disorder (ADHD), and the second has frontal lobe epilepsy. Both presented with attention problems, hyperactivity, and impulsivity associated with significant impairment in multiple settings. Two certified PCIT therapists provided 17 sessions to the parents of the first patient and 25 sessions to the parents of the second patient. Most of the sessions were in-person; however, some were “virtual” due to the circumstances associated with the COVID-19 pandemic. Parents of both patients achieved the “mastery” criteria. In both cases, PCIT contributed to improving the disruptive behaviors. PCIT may serve as an effective therapeutic option for young children with externalizing symptoms in Dubai.

## 1. Introduction (Background)

Attention-Deficit/Hyperactivity Disorder (ADHD) is a chronic neurodevelopmental disorder of childhood characterized by abnormally high levels of inattention and/or hyperactivity and impulsivity [1]. Children with ADHD have significant functional impairment in several settings including home, school, and in public [2]. About half of the children diagnosed with ADHD will experience the persistence of the disorder into adulthood [3]. Presence of comorbid oppositional defiant disorder predicted higher risk of persistence into adulthood [4].

ADHD effects about one in twenty children, across cultures [5]. However, nearly half to two-thirds of children with ADHD do not receive specialized services [6]. Therefore, effective treatment for individuals with this disorder is a major global health concern.

Children with ADHD commonly present with emotional dysregulation [7]. Dysfunction in brain circuits involved in processing of emotions has been reported [8]. Furthermore, deficits in some cognitive abilities, such as inhibitory functions and working memory, have been linked to externalizing symptoms [9]. According to a metanalysis of 44 studies, presence of oppositionality, externalizing behavior problems, and aggression predicted significantly higher levels of parenting stress [10]. Another metanalysis, which included 23 studies (1144 participants), indicated that PCIT was associated with significant reduction in externalizing behavior as well as parental stress [11].

Parent-child interaction therapy (PCIT) is available internationally for parents with children aged 2 to 7 years [11]. However, has been introduced to the Arab region recently, in 2019, with only three certified professionals at present. There has not been any studies on PCIT in the region so far.

PCIT is based on social learning and attachment theories and is designed as a parent training intervention [11]. The therapy's goal is to reduce the child's disruptive behaviors by improving the parent-child relationship [12]. The session begins with education followed by direct couching. The therapist observes the parent-child interaction and uses a bug-in-the-ear device to guide the parent. The parents are supported towards developing skills of attending positively, predictably, and consistently to the child's behaviors and play. The therapy has two phases: child-directed interaction (CDI) and parent-directed interaction (PDI) [12]. More information is available at http://www.pcit.org/.

In this case report, we describe the treatment course of PCIT for two young children treated in Dubai. The first child has been diagnosed with ADHD, and the second child had frontal lobe epilepsy with associated behavioral challenges. For the first child, we will use the pseudonym of Sami Rami, and for the second child, we will use the pseudonym of Anna John.

## 2. Case Presentation

### 2.1. Case 1

“Sami Rami” is a 5-year and 8-month-old boy from Arabic origin. He is a kindergarten student in a regular class at a governmental school. Sami lives with both parents and two siblings, his twin brother, and an 8-month-old brother.

Sami was assessed at the out-patient clinic by a consultant child and adolescent psychiatrist. He presented with his parents due to concerns related to hyperactivity and inattention, which started around 4 years of age. His poor concentration and forgetfulness impacted his academic performance. He was noted to be talkative, interrupted others during conversations, and had difficulties to sit when expected in the classroom. Furthermore, he struggled with impulsivity and emotion dysregulation resulting in fights with classmate on multiple occasions.

At 2 years of age, his aunt was babysitting him for about 3 months and on some occasions used physical punishment. Sami has met his motor, speech, social, and self-care developmental milestones. An evaluation based on DSM-5 diagnostic criteria [13] along with observation of his behavior and collateral history from his teacher revealed a diagnosis of attention-deficit/hyperactivity disorder (ADHD), combined presentation. He was prescribed with stimulant medication, methylphenidate immediate release 5 mg in the morning and 2.5 mg at noon to target his ADHD symptoms. Additionally, a referral was submitted to the PCIT Program.

A total of 17 PCIT sessions were offered. The plan was to conduct them on weekly basis. However, they were provided over an 8-month period due to interruptions caused by circumstances related to the COVID-19 pandemic. The therapy was offered by a PCIT-certified psychologist. The first phase, which was based on child-directed interaction (CDI), took place during 10 sessions. During this phase, Sami's parents were taught positive parenting skills as they were following their son's lead during the interaction.

Furthermore, training was provided on avoiding negative interactions, for example, criticism, and controlling commands. Furthermore, Sami's parents were guided to ignore inappropriate behavior. During the CDI phase, the therapist also educated Sami's parents about the PRIDE skills: P for praising the child, R for reflecting the child's behavior, I for imitating the child, D for describing their child's behavior, and E for using enthusiasm.

The second phase of PCIT was based on parent-directed interaction (PDI). This phase was provided virtually, instead of in-person due to the coinciding COVID-19 lockdown. It was provided over 7 consecutive weeks. During this phase of therapy, Sami's parents were trained to give effective commands. Furthermore, education was provided about utilizing the consequences protocol.

During the PCIT program, Sami's parents and the therapist discussed problematic behaviors as well as progress at the beginning of each session. Furthermore, the parents recorded data about Sami's behavior on a timetable during the week and discussed it with the therapist during the session.

Upon the completion of 17 PCIT sessions, Sami's parents met criteria for “mastery.” They indicated that they have noted a significant decline in the frequency, duration, and intensity of externalizing symptoms, for instance, behavioral outbursts. Additionally, he became more cooperative in following his parents' instructions. Furthermore, his play pattern with peers and siblings became respectful. In regard to his activity level, it was noted to become more regulated early during the course of treatment, and this coincided with the initiation of treatment with methylphenidate medication.

### 2.2. Case 2

“Anna John” is a 6-year and 8-month-old girl from a South American origin. She is a kindergarten student at a private school. She lives with her parents and 9-year-old sister.

Anna followed at the neurology clinic since early childhood. She was diagnosed with frontal lobe epilepsy at 4 months of age and was managed with antiepileptic medications, oxcarbazepine, and clobazam. She was diagnosed with dysphasia at 2 years of age. Later, she was diagnosed with intellectual disability and had a total intelligence quotient (IQ) score of 65. She presented with her parents to the psychiatry clinic for concerns related to hyperactivity, emotion dysregulation, and aggressive behavior. Additionally, she had academic challenges as well as expressive and receptive language difficulties.

Anna had behavioral outbursts in the form of screaming and throwing things around. There were precipitated by instructions on how to do things correctly, for instance, wearing her shoes in the correct foot. Her parents also commented on hyperactivity in the form of running around and climb on furniture. She was noted to be talkative and often interrupted others during conversations. Additionally, she struggled with inattention, and she got easily bored.

In regard to developmental milestones, Anna had delays in multiple domains, including motor skills, speech, and language, as well as socially. The evaluation conducted by a consultant child, and adolescent psychiatrist revealed that her presentation is likely explained by her underlying intellectual challenges and epilepsy. Executive dysfunction, cognitive impairment, and hyperactivity have been reported in individuals diagnosed with frontal lobe epilepsy [14]. Medication to target her hyperactivity and inattention were not prescribed. A referral was submitted to the PCIT program.

A total of 25 PCIT sessions were offered. The plan was to conduct them on weekly basis. However, there was a 7-month gap, after the fifth session, due to interruptions caused by circumstances related to the COVID-19 pandemic. The therapy was offered by a PCIT-certified psychologist. The first phase, which was based on child-directed interaction (CDI), took place during 15 sessions. The last 4 sessions of this phase were provided virtually instead of in-person. During this phase, Anna's parents were taught positive parenting skills as they were following their daughter's lead during the interaction.

Furthermore, training was provided on avoiding negative interactions, for example, criticism, and controlling commands. Furthermore, Anna's parents were guided to ignore inappropriate behavior. During the CDI phase, the therapist also educated the parents about the PRIDE skills.

The second phase of PCIT was based on parent-directed interaction (PDI). The first 3 sessions during this phase was provided virtually, instead of in-person due to the coinciding COVID-19 lockdown. The remaining 7 sessions were provided in-person. The second phase was provided over 10 consecutive weeks. During this phase of therapy, Anna's parents were trained to give effective commands. Furthermore, education was provided about utilizing the consequences protocol.

During the PCIT program, Anna's parents and the therapist discussed problematic behaviors as well as progress at the beginning of each session. Furthermore, the parents recorded data about Anna's behavior on a timetable during the week and discussed it with the therapist during the session.

Upon the completion of 25 PCIT sessions, Anna's parents met criteria for “mastery.” They indicated that they have noted a significant decline in the frequency, duration, and intensity of externalizing symptoms, for instance, tantrums. Additionally, her parents noted improved in the quality of their relationship with Anna. Furthermore, Anna's parents commented on improvement in attention span and engagement in tasks for longer periods.

## 3. Discussion

Two cases of inattention, hyperactivity, and impulsivity were illustrated. The first case had a diagnosis of ADHD, while the second had frontal lobe epilepsy. PCIT resulted in positive effect in reducing externalizing behaviors in both cases. Hyperactivity and attention improved in the first case in response to stimulant medication treatment. Hyperactivity symptoms persisted in the second case; however, attention improved, and she was able to engage in activities for longer durations. Parents in both cases experienced improvement in the quality of their relationship with their children.

The results of this case report are in keeping with previous studies, in regard to its positive effect on externalizing behaviors as well as interpersonal relationships [11]. Furthermore, efficacy was noted upon parents achieving the criteria for “mastery” [15]. In contrast to previous studies, some sessions were conducted virtually. In addition, due to the COVID-19 pandemic circumstances, there were relatively long gaps between some of the sessions.

From a social learning perspective, PCIT enhances desired behaviors by providing positive reinforcement [16], as per the PRIDE skills. Furthermore, it diminishes undesired behaviors by intentionally ignoring these behaviors [17]. For case 1, this corresponded to playing with siblings and peers “calmly” opposed to playing roughly. For case 2, this corresponded to speaking with relatives “politely” opposed to screaming. Giving consistent instructions and being predictable in responding to behaviors enabled the children to have more clarity on expectations. From an attachment perspective, the parent's engagement with enthusiasm has probably contributed to enhanced emotional regulation in their children [18].

Although externalizing symptoms in neurodevelopmental disorders are explained by underlying biological dysfunction [19, 20], it seems that PCIT's role in enhancing parenting skills can have a significant positive impact.

## 4. Conclusion

The two reported cases indicate that PCIT seems to be effective in improving externalizing behaviors among young children in Dubai. Future research using randomized controlled trails is recommended to confirm the consistency of these results. Furthermore, follow-up studies will assist in determining how long the therapy effects last, after the completion of the treatment course.

## Data Availability

Data used to support the findings of this study are included within the article.
